# One‐Stage Posterior‐to‐Anterior Ankle Arthroscopy Using a Single Operative Setup and Patient Repositioning Technique: A Technical Note

**DOI:** 10.1002/atn2.70380

**Published:** 2026-11-13

**Authors:** Julien Paquot, David Roche, Ronny Lopes

**Affiliations:** ^1^ Centre Orthopédique Santy, FIFA Medical Center of Excellence Hôpital Privé Jean Mermoz, Groupe Ramsay Santé Lyon France

## Abstract

This technical note describes a 1‐stage arthroscopic technique for the management of combined posterior and anterior ankle pathology that preserves standard patient positioning while reducing operative time and material costs. The procedure begins with posterior ankle arthroscopy performed in the prone position using standard posterior portals. After treatment of the posterior lesions, the patient is carefully repositioned to the supine position without redraping or changing instrumentation. The same sterile surgical field, operating table, and arthroscopic instruments are maintained throughout the procedure. Anterior ankle arthroscopy is then performed using standard anterior portals. Strict coordination between the surgical and anesthesia teams ensures safe patient repositioning while preserving sterility. This 1‐stage technique allows arthroscopic treatment of combined anterior and posterior ankle pathology using familiar prone and supine positioning. By avoiding redraping and the use of duplicate sterile instrumentation, it optimizes surgical workflow, reduces operative time, and decreases material costs while providing a reproducible approach.

## Graphical abstract

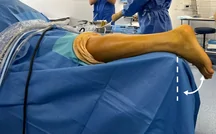

**VIDEO 1:** One‐stage posterior‐to‐anterior arthroscopy of the left ankle. The patient is initially positioned prone (phase A) for posterior ankle arthroscopy through standard posterior portals. Without removing the sterile drapes or arthroscopic instrumentation, the patient is then carefully repositioned to supine (phase B) for anterior ankle arthroscopy through standard anterior portals using the same operating table and sterile field throughout. Video content can be viewed at https://doi.org/10.1002/atn2.70380.

Ankle arthroscopy is a well‐established technique that provides reliable pain relief and functional improvement.^1^ Safe anterior and posterior arthroscopic approaches have been described to address pathologies of the tibiotalar joint from either compartment.^2^, ^3^, ^4^ However, some patients present with combined anterior and posterior lesions for which conservative treatment has failed, requiring surgical intervention. In these cases, the surgeon must choose between a 2‐stage procedure and a single‐stage approach. Traditional positioning limits comprehensive access to both compartments because the anterior approach in the supine position does not allow adequate posterior visualization, and the prone position presents the opposite limitation. Although 1‐stage strategies have been reported, considerable variation persists in how patient positioning is managed. The purpose of this technical note is to describe a streamlined 1‐stage arthroscopic technique that begins with posterior ankle arthroscopy in the prone position, followed by a controlled transition to the supine position without redraping to complete the anterior arthroscopy.

## SURGICAL TECHNIQUE

A 1‐stage arthroscopic procedure is performed, beginning with posterior ankle arthroscopy with the patient in the prone position, followed by repositioning to the supine position for anterior ankle arthroscopy while maintaining the same sterile draping, instruments, and operating table throughout the procedure (Video 1).

### Patient Setup

The patient is positioned so that the foot and ankle extend beyond the edge of the operating table, allowing full ankle dorsiflexion and plantarflexion throughout the procedure (Figure 1). A thigh tourniquet and an arthroscopy pump may be used at the surgeon's discretion. Standard preoperative antibiotic prophylaxis is administered.

**FIGURE 1:**
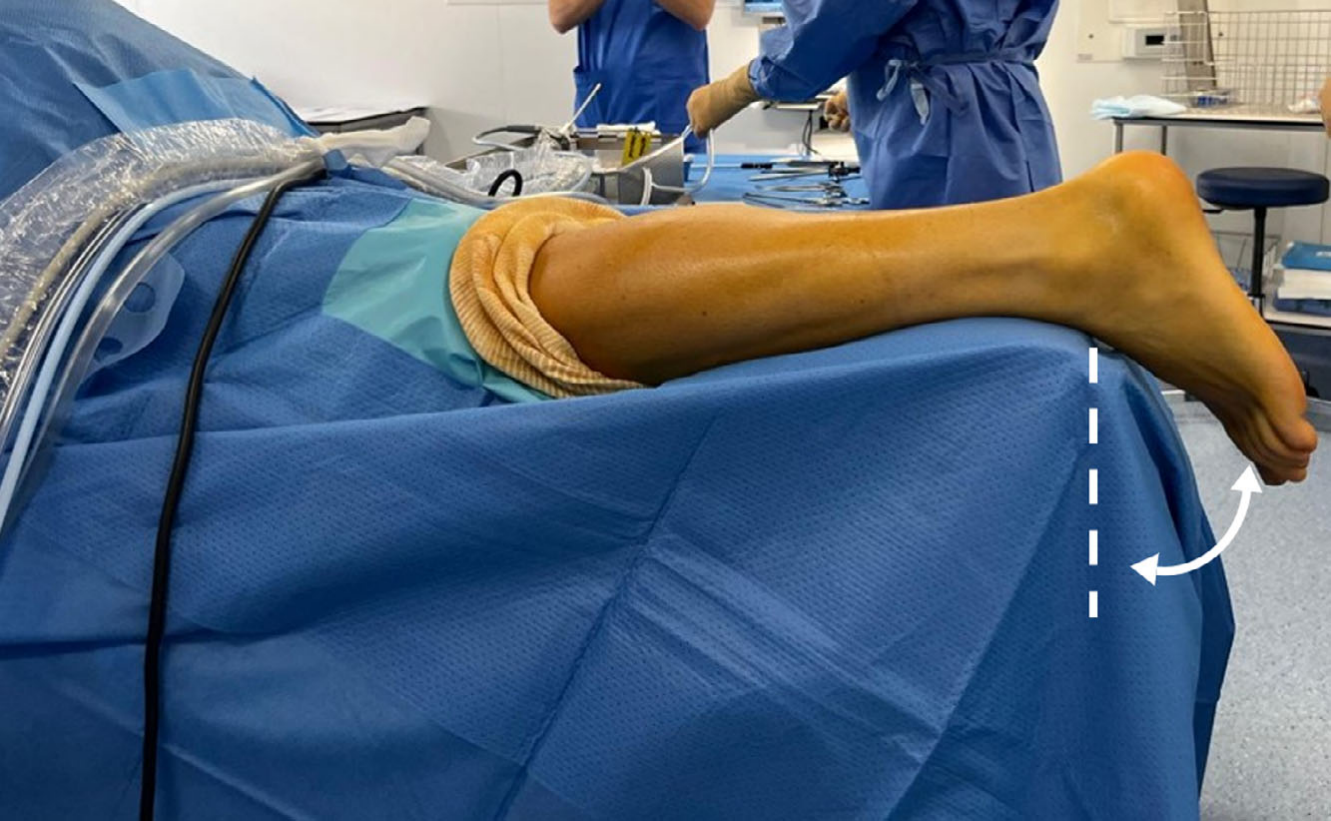
Patient positioning for 1‐stage posterior‐to‐anterior arthroscopy of the left ankle, shown in the prone position. The operative foot and ankle are positioned beyond the distal edge of the operating table, allowing unrestricted dorsiflexion and plantarflexion throughout both the posterior and anterior stages of the procedure.

### Posterior Ankle Compartment Arthroscopy

Under general or spinal anesthesia, the operative limb is prepared and draped in a standard sterile fashion with the patient in the prone position. The arthroscopy monitor is positioned on the same side as the operative limb. The remaining arthroscopic equipment, including the camera, shaver, and associated cables, is placed on the side of the nonoperative leg on a table positioned perpendicular to the operating table, which facilitates equipment organization and reduces the risk of breaches in sterility. All cables are routed over the patient's limb to avoid interference with the surgical field (Figure 2). Standard posterior ankle arthroscopic portals are then established, allowing diagnostic exploration and treatment of posterior ankle pathology as indicated. On completion of the posterior procedure, the portals are closed with sutures, and a sterile dressing is applied to protect the wounds before patient repositioning.

**FIGURE 2:**
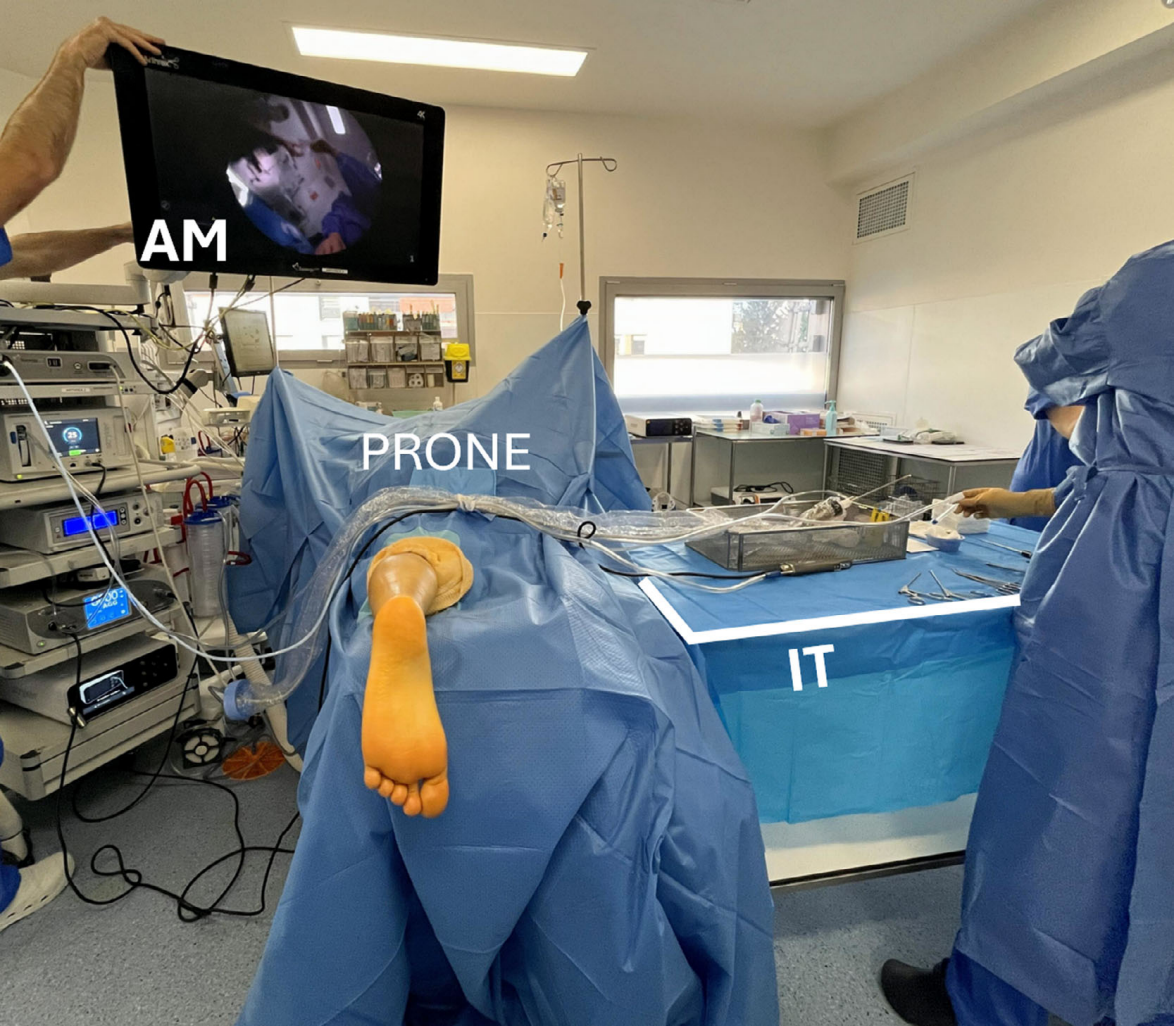
Operating room setup for posterior arthroscopy of the left ankle, patient prone. The AM is positioned on the same side as the operative limb. An IT is placed perpendicular to the operating table on the nonoperative leg side, and all cables are routed over the operative limb rather than across the surgical field, reducing contamination risk and keeping the workspace organized during the posterior‐compartment stage. (AM, arthroscopy monitor; IT, instrument table.)

### Patient Repositioning

Before repositioning, the anesthesia team confirms that all monitoring devices and airway management equipment are securely fixed. All arthroscopic instruments and cables are removed from the surgical field and maintained in a sterile condition on the operative table positioned adjacent to the arthroscopy monitor. Patient repositioning requires strict coordination and involves 2 teams, the surgical team and the anesthesia team, typically comprising 5 to 6 personnel. Each team member is assigned a defined role to ensure patient safety and preservation of sterility. Within the surgical team, 1 member supports the lower limbs, whereas another maintains the sterile drapes in a vertical position to provide access to the patient's body for the anesthesia team. Within the anesthesia team, 1 member supports the pelvis, another stabilizes the thorax, and a final member secures the head and neck (Figure 3).

**FIGURE 3:**
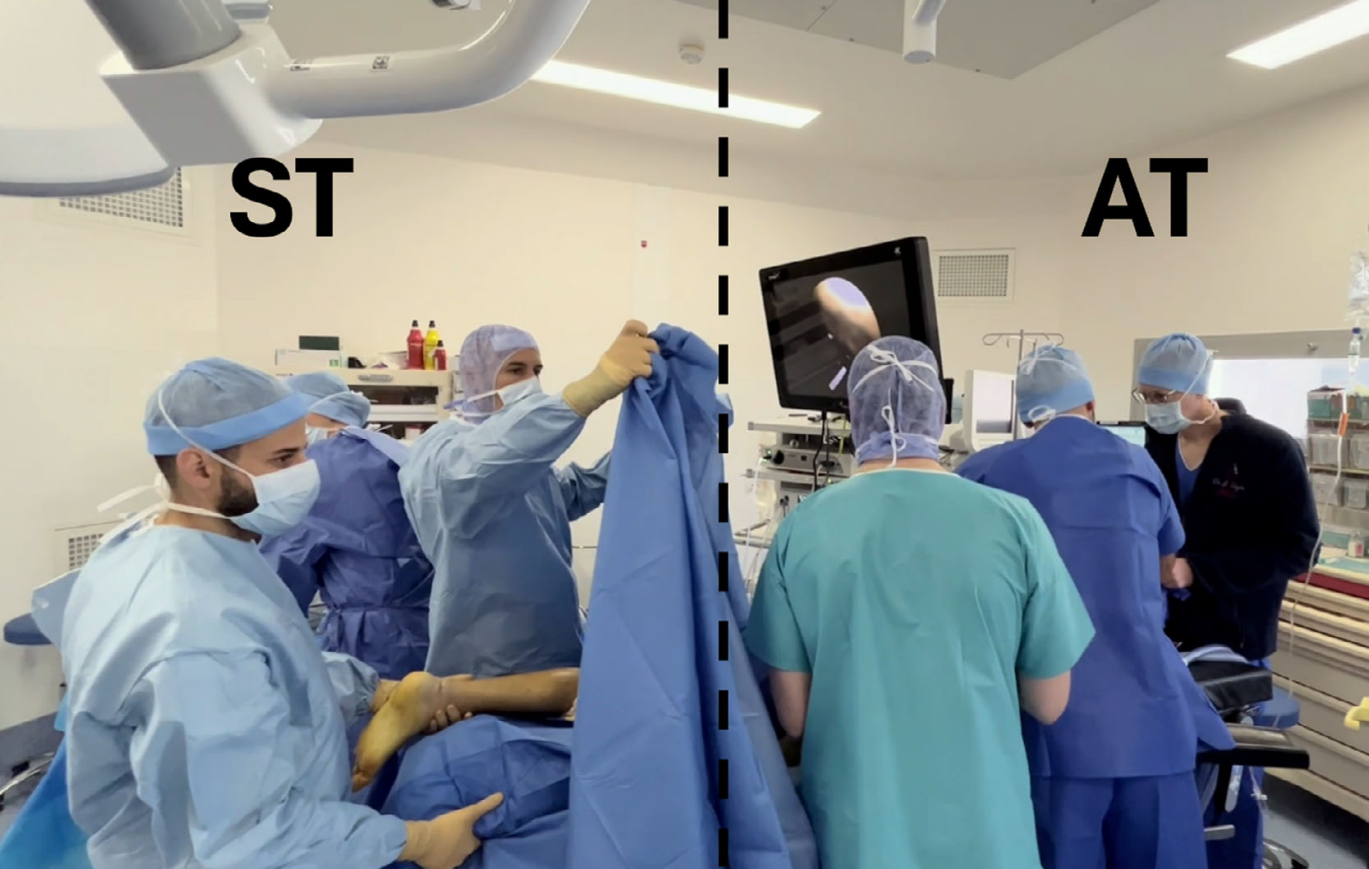
Coordinated intraoperative repositioning of the patient from prone to supine during 1‐stage posterior‐to‐anterior arthroscopy of the left ankle. The ST (left) maintains the sterile drape vertically and supports the lower limbs, whereas the AT (right) simultaneously supports the pelvis, thorax, and head/neck. This synchronized 2‐team maneuver (5‐6 personnel) allows safe patient turning without breaking sterility or removing drapes between the posterior and anterior stages. (AT, anesthesia team; ST, surgical team.)

Under coordinated supervision, the patient is carefully turned from the prone to the supine position, ensuring that the operated leg is positioned over the nonoperated leg. All pads used for prone positioning are then removed to allow safe and appropriate supine positioning.

### Anterior Ankle Compartment Arthroscopy

After repositioning, the arthroscopy equipment is rearranged for a standard anterior ankle arthroscopy setup. Standard anterior ankle portals are established, allowing diagnostic exploration and treatment of anterior ankle compartment pathology, as indicated (Figure 4). At the conclusion of the procedure, the anterior portals are closed with sutures, and sterile dressings are applied.

**FIGURE 4:**
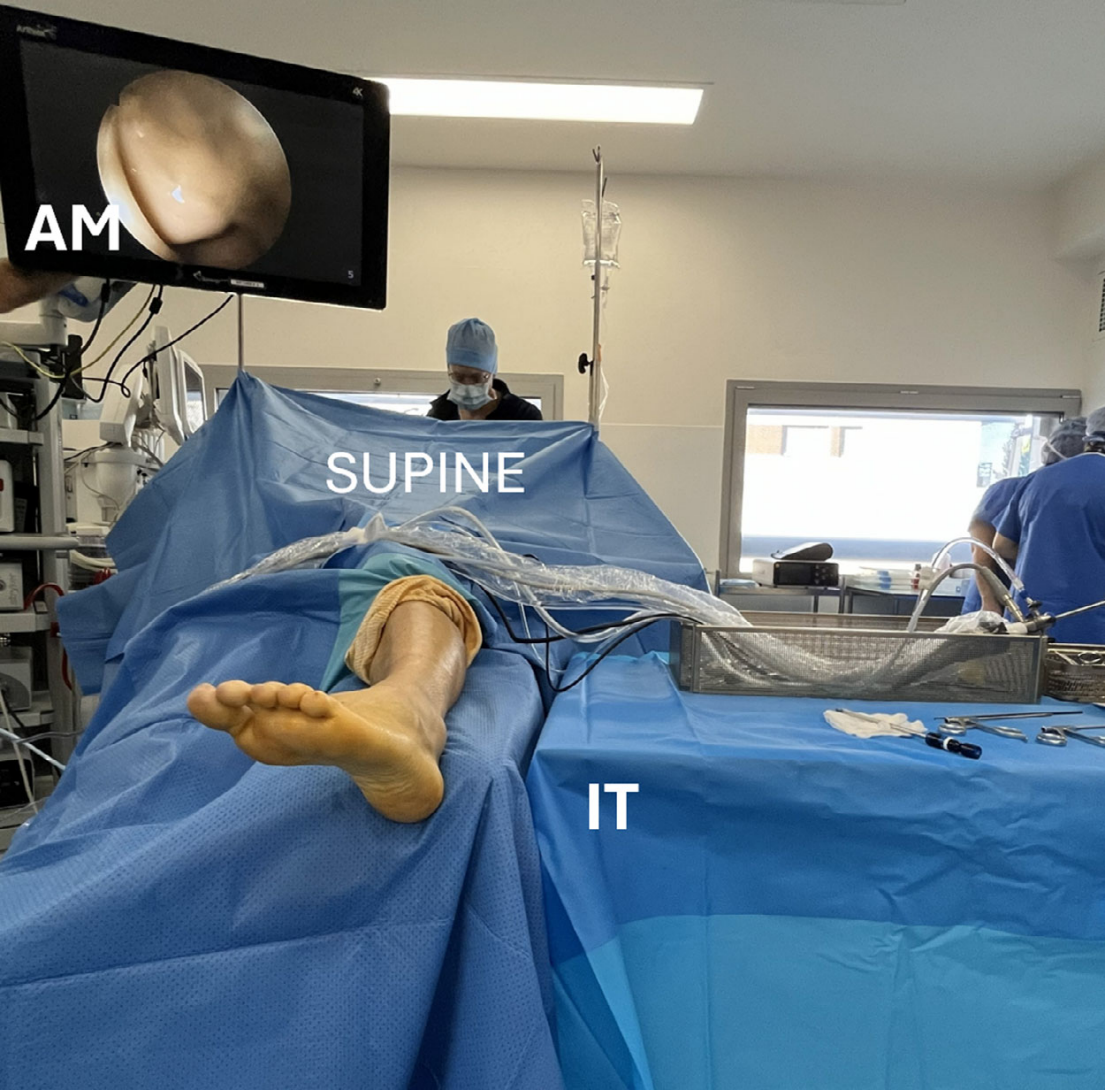
Operating room setup for anterior arthroscopy of the left ankle after repositioning to supine. The same AM and IT, with the same sterile instrument tray used during the posterior stage, are reused without redraping. The operative foot is again positioned beyond the table edge, allowing standard anterior portal placement. (AM, arthroscopy monitor; IT, instrument table.)

### Ethical Approval and Consent

This technical note does not involve human or animal experimentation. Informed consent was obtained from the patient for the use of intraoperative images and video material for publication.

## DISCUSSION

This technical note describes a 1‐stage ankle arthroscopic procedure beginning with posterior ankle arthroscopy performed in the prone position, followed by repositioning the patient to the supine position without redraping while maintaining the same sterile field, operating table, and sterile instruments for the anterior arthroscopic procedure. This sequence allows the surgeon to use standard and familiar positioning for both posterior and anterior ankle arthroscopy.

Several alternative techniques have been described for the combined arthroscopic management of the anterior and posterior ankle compartments, including approaches based on modified knee or hip positioning or posterior arthroscopy performed in the supine position using specific portals.^5^, ^6^, ^7^, ^8^ Although effective, these approaches often rely on nonstandard positioning, altered anatomical orientation, or the use of traction, which may complicate the procedure, limit surgical ergonomics, and, as highlighted by Rodríguez‐Merchán et al. increase the risk of neurologic complications.^9^

The technique presented in this technical note combines the advantages of standard prone and supine positioning without the need for redraping or resterilization. Maintaining the same sterile field, operating table, and instruments throughout the procedure simplifies surgical workflow, reduces operative time and costs, and minimizes the risk of contamination while providing a reproducible solution for the arthroscopic treatment of concomitant anterior and posterior ankle pathology.

The advantages and limitations of this technique are summarized in Table 1, and key technical pearls and pitfalls are presented in Table 2.

**TABLE 1:** Advantages and Limitations of 1‐Stage Management of Anterior and Posterior Arthroscopy

**Advantages**	**Limitations**
• Advantages from standard patient positioning for anterior and posterior arthroscopy • Standard anatomical landmarks • Decreased costs without redraping the patient • Decreased surgical time • Surgical team remains sterile throughout procedure	• Team training • Team coordination • Five to 6 team members for patient repositioning and maintenance of sterile instrumentation • Theoretical risk of contamination

**TABLE 2:** Our Pearls and Pitfalls Regarding the 1‐Stage Management of Anterior and Posterior Arthroscopy

**Pearls**	**Pitfalls**
• Placing the slide sheet before patient positioning on the surgical table facilitates the patient repositioning • Tourniquet air‐inflow should be on the lateral side of the thigh • Cross the feet before turning the patient • The patient repositioning should be done synchronously • The proximal part of the sterile drape is kept proximal to the surgical field to prevent contamination	• The absence of the slide can lead to improper synchronization of the repositioning process • Poor communication and synchronization between teams may complicate patient repositioning

This technique requires strict coordination between the surgical and anesthesia teams during patient repositioning and may require additional personnel. Although patient repositioning carries a theoretical risk of contamination, this risk can be minimized with a trained team and an adequately sized operating room. The transition time between posterior portal closure and anterior portal incision averaged 4 minutes. The reported cost savings reflect the economic conditions of our institution and may vary depending on institutional practices, health care systems, and country‐specific cost structures.

This 1‐stage technique reduces the operative time and material consumption compared with the conventional 2‐stage approach. By maintaining a single sterile field and arthroscopic instrumentation, duplication of equipment is avoided, resulting in an estimated material cost saving of approximately $157 per procedure (Table 3) while preserving standard prone and supine positioning and ergonomic working conditions.

**TABLE 3:** Estimated Material Cost Savings Achieved by Avoiding Redraping Between Posterior and Anterior Ankle Arthroscopy

**Material**	**Cost, $**
• Ankle arthroscopy pack (including drapes, compresses, and tubing)	78.30
• Suction tubing	0.68
• Sterilization of arthroscopy ancillary instruments	21.00
• Shaver blade	57.00
Total cost saved per procedure	156.98

## DISCLOSURES

The author (R.L.) declares the following financial interests/personal relationships which may be considered potential competing interests: R.L. reports grants or contracts from Arthrex, Steps Ortho, and Enovis; royalties or licenses from Steps Ortho and Enovis; consulting fees from Arthrex, Steps Ortho, and Enovis; payment or honoraria for lectures, presentations, speakers bureaus, manuscript writing, or educational events from Arthrex and Steps Ortho; and participation on a data safety monitoring board or advisory board from ANKLE GO. The other authors (J.P., D.R.) declare that they have no known competing financial interests or personal relationships that could have appeared to influence the work reported in this paper.
